# Chest Drain Size: the Debate Continues

**DOI:** 10.1007/s13665-017-0162-3

**Published:** 2017-01-26

**Authors:** Robert J. Hallifax, Ioannis Psallidas, Najib M. Rahman

**Affiliations:** Oxford Centre for Respiratory Medicine, Oxford University Hospitals NHS Trust, Churchill Hospital, Oxford, OX3 7LE UK

**Keywords:** Chest drain, Small bore, Size, Pleural, Effusion, Pneumothorax, Pleural Infection

## Abstract

**Purpose of review:**

Small-bore chest tubes are widely used in the management of common pleural disease. Guidelines suggest that patients with malignant pleural effusions, pneumothorax and pleural infection may be successfully managed with small-bore drains. However, good quality data is often lacking. This article reviews the evidence for the treatment efficacy and potential adverse effects of different chest tube sizes.

**Recent findings:**

In a large randomised study, the small difference in pain scores between large and small drains was not clinically significant. However, small-bore chest tubes commonly suffer from blockage or inadvertent removal, and may not be as effective in providing successful pleurodesis for malignant pleural effusions.

**Summary:**

Although they may be effective in managing pleural infection, and less painful than large drains, small bore drains may be less effective for pleurodesis.

## Introduction

The optimal size of chest tube for management of pleural diseases is not known. Management of malignant pleural effusions traditionally relies on chest tube insertion, drainage of fluid and subsequent talc pleurodesis. The British Thoracic Society (BTS) guidelines [1] advocates the use of smaller tubes (<16 French [F]). The evidence for smaller tubes is based upon case series [1] and three comparative studies [2–4], of which only one small study was randomised [4].

Patients who are symptomatic with a large spontaneous pneumothorax often require chest tube insertion, after attempted aspiration in primary spontaneous pneumothorax. Guidelines suggest that, in clinically stable patients, small-bore (≤14 F) chest drains are sufficient [1, 5]. The management of pleural infection requires drainage of the infected fluid. Traditionally, larger bore chest tubes have been used to drain pus or viscid fluid. However, small-bore chest drains are often the first line of choice of radiologists and respiratory physicians and may be sufficient in many cases. This article will review the evidence for the treatment efficacy and potential adverse effects of different chest tube sizes for these indications.

## Efficacy

### Pleurodesis for Malignant Pleural Effusion

The most commonly used agent for pleurodesis to prevent fluid recurrence in malignant pleural effusions in the USA and Europe is sterile graded talc. At present, standard practice for talc pleurodesis involves instillation of talc slurry via chest tube (once the pleural fluid has been drained). Success rates for talc pleurodesis are quoted in the BTS guidelines as between 60 and 90% [1]. The same guidelines advocate the use of small-bore chest tubes. However, 7 of the 15 studies cited in the guidelines used large-bore (20–28 F) chest tubes as was historically often used [1]. The evidence for efficacy in small-bore tubes comes from case series, two retrospective comparative studies [2, 3] and only one small randomised study of 18 patients which was a feasibility study, not designed to compare efficacy [4].

The recently published TIME1 (1st Therapeutic Interventions in Malignant Effusion) randomised controlled trial (RCT) assessed the effect of chest tube size and analgesia (NSAIDs vs opiates) on pain and clinical efficacy related to pleurodesis in patients with malignant pleural effusion [6••]. Designed as a non-inferiority study (with a margin of 15%), the authors found that 12 F chest tubes were associated with higher pleurodesis failure rate than 24 F tubes (30 vs 24%), thereby failing to meet non-inferiority criteria (difference, −6%; one-sided 95%CI, −20% to ∞; *p* = 0.14). However, a large number (206) of the patients recruited in the study had undergone a thoracoscopy (in which a large-bore drain is always placed) and so could not be included in the primary comparison for pleurodesis efficacy by drain size. Nevertheless, the 100 patients included in this analysis still represent the largest study to address this question, from which we cannot conclude equivalence between small- and large-bore drains.

An alternative approach to talc pleurodesis is the poudrage (or insufflation) of talc powder at thoracoscopy. A Cochrane review (2004) found that talc poudrage at thoracoscopy to have an improved relative risk or non-recurrence (1.19) compared to talc slurry [7]. However, a later RCT suggested only a trend towards superiority of talc poudrage and only in a sub-group analysis (excluding those patients with trapped lung) did the authors find a statistically significant difference [8]. Another large non-randomised prospective study found fewer pleurodesis failures in the talc poudrage group. As a result of these conflicting data, an RCT to directly compare talc poudrage to talc slurry for pleurodesis efficacy is currently recruiting in the UK [9]. Although designed to directly compare poudrage vs slurry, this ongoing study may be hampered in its conclusions by the result of the TIME1 study [6••] as the patients in the post-thoracoscopy arm will have large-bore chest drains and those receiving slurry small-bore drains, which may not be as effective as per the TIME1 result.

### Pneumothorax

Patients who are symptomatic with large spontaneous pneumothorax often require chest tube insertion. The BTS and the Belgian Society of Pneumology Guidelines suggest attempted needle aspiration first, followed by chest drain if the lung has failed to sufficiently reinflate [1, 10]. The American College of Chest Physicians (ACCP) guidelines advise initial treatment with chest tube, although these may now be out of date (2001) [5]. All guidelines suggest that in clinically stable patients, small-bore (≤14 F) chest drain are sufficient [1, 5, 10]. There exists no randomised trial data. The majority of the evidence for these guidelines comes from multiple case series [11]. One small retrospective comparative study found no difference in treatment success between large-bore (20 F) and small-bore (9 F) chest drain [12].

Failure of treatment of spontaneous pneumothorax is typically defined as the need for further procedure (i.e., another chest tube) or surgical intervention, if the lung fails to re-expand or there is persistent air leak. Suction delivered via high-volume low-pressure system can be employed in such patients. Some physicians may also advocate replacing a small-bore tube with a large-bore one to allow a greater total flow rate of air—as flow rate is proportional to the fifth power of the radius of the tube (Fanning equation). The theory in both cases is that the air can be removed from the pleural cavity at a rate exceeding the egress of air through the breach in the visceral pleura, thereby reinflating the lung. This theory would seem rational if there is clear evidence of insufficient drainage of air (i.e., subcutaneous emphysema, increasing pneumothorax size, or worsening clinical condition). However, there is no good trial evidence to support the use of larger drains or suction in stable patients with a continuing air leak. The idea that the “healing” of the visceral air leak may be promoted by apposition of the visceral and parietal pleural layers has not been proven.

A large-bore chest tube (>28 F) is often recommended in the management of traumatic pneumothorax given the potential need of air and/or blood evacuation. However, this is based upon expert opinion [13] and a recent small RCT, in fact, found that for simple, uncomplicated traumatic pneumothorax, use of a small chest tube (14 F) was associated with reduced pain at insertion and no other clinically important differences (such as drain duration or success rate) [14•].

### Pleural Infection

Pleural infection carries a high mortality and 15% large proportion of patients will require surgical intervention [15]. Effective management of pleural infection requires drainage of the infected fluid. Traditionally, larger bore chest tubes have been used to drain pus or viscid fluid. However, numerous case series have reported successful treatment with small-bore chest drains which are often the first line choice of radiologists and respiratory physicians. A largest prospective RCT of pleural infection included 405 patients treated with a range of chest tube sizes [15]. Patients were randomised to intra-pleural fibrinolytics or placebo, rather than small (≤14 F) or larger chest tube, but clinical outcomes, pain, and adverse events could be analysed by chest tube size [16]. The authors found no significant difference in the frequency with which patients either died or required thoracic surgery between groups with chest tubes of varying sizes (<10 F, number dying or needing surgery 21/58 (36%); size 10–14 F, 75/208 (36%); size 15–20 F, 28/70 (40%); size >20 F, 30/69 (44%); *p* = 0.27). It was concluded that smaller chest tubes could be as effective as large bore and may therefore be the initial treatment of choice for pleural infection. A subsequent randomised study of pleural infection found improved fluid drainage and reduced the frequency of surgical referral in the patients receiving tissue plasminogen activator and DNase (compared to placebo). The majority of these patients were managed with small-bore drains (<15 F) [17].

## Adverse Events

The complication rate for chest drain insertion is regularly retrospectively collected as part of the BTS Pleural Procedures Audit. The 2015 audit included 1394 episodes of chest drain insertion, the majority of which (88%) were small bore (<16 F) [18•]. Complications were categorised as immediate or delayed. Immediate complications were failure to place the drain in the pleural space (2.0%), haemothorax (iatrogenic) 1.3%, pain (8%), hypotension (1.9%) and organ puncture (0.6%). Delayed complications were pain (15.6%), drain falling out (9.2%), drain blockage (8.2%), subcutaneous emphysema (4.2%), pleural infection (0.4%), skin infection (1%), re-expansion pulmonary oedema (0.6%) and death (0.1%). A retrospective review of 100 consecutive patients who underwent small-bore chest tube placement found that complications occur less frequently if the drain is placed by expert operators or under radiological/ultrasonographic guidance [19]. Use of ultrasound in chest drain insertion of pleural fluid is now mandatory, but only 70% of chest drains and pleural aspirations were performed using ultrasound guidance in the recent BTS audit [18•].

### Pain

Two randomised studies have suggested that large-bore chest tubes may be associated with greater pain. The first was a small (18 patients) feasibility study in patients undergoing tetracycline pleurodesis [4]. The second was a larger (100 patients) study of patients post-thoracic surgery which compared “small” (19 F) drains with standard larger drains (28–32 F) [20].

Analysis of the pain scores in 128 patients in the MIST1 study of pleural infection also found that large bore tubes caused more pain, which was statistically and clinically significant. During chest tube insertion and while the tube was in place, 22/41 (54%) of patients with large bore tube (≥15 F) described moderate/severe pain, compared with only 21/77 (27%) of patients treated with small tube (<15 F) (*p* = 0.005) [16]. The authors conclude that “27% of patients with larger-size tubes experienced moderate to severe pain that might have been avoided by use of small-size-tubes”.

The larger TIME1 study was undertaken to directly assess pain as a co-primary outcome by chest tube size and analgesia type for malignant effusions [6••]. Data from 300 patients found a statistically significant difference in pain scores between smaller and larger tubes. However, this, on average, was small (6 mm) and was below the published minimum clinically significant threshold for a 100 mm VAS pain score (13 mm; 95%CI, 10–16 mm) [21]. This difference was consistent even when accounting for rescue analgesia usage. Therefore, there would appear to be relatively little clinical benefit in terms of less pain from the use of smaller chest tubes for malignant pleural effusion pleurodesis.

### Complications Post-insertion

Small-bore chest tubes appear to be at greater risk of blockage, kinking, or inadvertent removal. Older data suggested a blockage rate of small-bore tubes of 8.1% compared to 5.2% for large-bore tubes in a prospective (non-randomised) study [22]. Recent data suggests a non-significant increase in rate of complications during tube insertion in the small (12 F) group compared to 24 F group (25 vs 14%, *p* = 0.20) and a higher rate of unintentional displacement of the tube (42 vs 28%) [6••]. Analysis of chest tubes used to treat found a trend towards an increased rate of unplanned chest tube displacement in the smaller tubes (<10 F, 19%; 10–14 F, 23%; size 15–20 F, 0%; >20 F, 17%), but this did not reach statistical significance (*p* = 0.18) [16]. The complication rates in data collected prospectively in RCTs are higher than those reported in the retrospective BTS audit.

## Conclusion

Guidelines suggest that patients with pneumothorax requiring chest tube insertion should be managed with small (rather than large) bore tubes. However, there is a dearth of evidence to support this. Non-RCT data suggests that pleural infection may be successfully initially managed with small bore drains. However, it is now unclear whether small bore chest tubes are as effective as larger drains for pleurodesis for malignant effusions. Large-bore tubes are often regarded being more painful, but recent RCT data now suggested that the small difference in pain scores are not clinical significant in patients with malignant effusion.

Complications in small-bore chest tubes (blockage or inadvertent removal) are higher than larger tubes, and may account for some of the reduced rate of successful pleurodesis. This may be an inherent disadvantage of smaller tubes. Use of small-bore chest tubes in the management of common pleural disease is ubiquitous [18•]. Discerning physicians will need to decide whether to alter their practice with regards to the optimal chest tube size for pleurodesis success.
